# Molecular surveillance of *Plasmodium vivax dhfr *and *dhps *mutations in isolates from Afghanistan

**DOI:** 10.1186/1475-2875-9-75

**Published:** 2010-03-14

**Authors:** Sedigheh Zakeri, Mandana Afsharpad, Faezeh Ghasemi, Ahmad Raeisi, Najibullah Safi, Waqar Butt, Hoda Atta, Navid D Djadid

**Affiliations:** 1 Malaria and Vector Research Group (MVRG), Biotechnology Research Center, Institut Pasteur of Iran, Pasteur Avenue, P.O. BOX 1316943551, Tehran, Iran; 2 National Programme Manager for Malaria Control, Ministry of Health and Medical Education, Tehran, Iran; 3 National Malaria and Leishmaniasis Control Programme Manager, Ministry of Public Health, Kabul, Afghanistan; 4 Medical Officer, Malaria and Leishmaniasis, WHO Office, Kabul, Afghanistan; 5 Roll Back Malaria, WHO/EMRO, Cairo, Egypt

## Abstract

**Background:**

Analysis of dihydrofolate reductase (*dhfr*) and dihydropteroate synthase (*dhps*) mutations in *Plasmodium vivax *wild isolates has been considered to be a valuable molecular approach for mapping resistance to sulphadoxine-pyrimethamine (SP). The present study investigates the frequency of SNPs-haplotypes in the *dhfr *and *dhps *genes in *P. vivax *clinical isolates circulating in two malaria endemic areas in Afghanistan.

**Methods:**

*P. vivax *clinical isolates (n = 171) were collected in two different malaria endemic regions in north-west (Herat) and east (Nangarhar) Afghanistan in 2008. All collected isolates were analysed for SNP-haplotypes at positions 13, 33, 57, 58, 61, 117 and 173 of the *pvdhfr *and 383 and 553 of the *pvdhps *genes using PCR-RFLP methods.

**Results:**

All 171 examined isolates were found to carry wild-type amino acids at positions 13, 33, 57, 61 and 173, while 58R and 117N mutations were detected among 4.1% and 12.3% of Afghan isolates, respectively. Based on the size polymorphism of *pvdhfr *genes at repeat region, type B was the most prevalent variant among Herat (86%) and Nangarhar (88.4%) isolates. Mixed genotype infections (type A/B and A/B/C) were detected in only 2.3% (2/86) of Herat and 1.2% (1/86) of Nangarhar isolates, respectively. The combination of *pvdhfr *and *pvdhps *haplotypes among all 171 samples demonstrated six distinct haplotypes. The two most prevalent haplotypes among all examined samples were wild-type (86%) and single mutant haplotype I_13_P_33_F_57_S_58_T_61_**N **_117_I_173/_A_383_A_553 _(6.4%).

Double (I_13_P_33_S_57_**R**_58_T_61_**N**_117_I_173_/A_383_A_553_) and triple mutant haplotypes (I_13_P_33_S_57_**R **_58_T_61_**N**_117_I_173_/**G**_383_A_553_) were found in 1.7% and 1.2% of Afghan isolates, respectively. This triple mutant haplotype was only detected in isolates from Herat, but in none of the Nangarhar isolates.

**Conclusion:**

The present study shows a limited polymorphism in *pvdhfr *from Afghan isolates and provides important basic information to establish an epidemiological map of drug-resistant vivax malaria, and updating guidelines for anti-malarial policy in Afghanistan. The continuous usage of SP as first-line anti-malarial drug in Afghanistan might increase the risk of mutations in the *dhfr *and *dhps *genes in both *P. vivax *and *Plasmodium falciparum *isolates, which may lead to a complete SP resistance in the near future in this region. Therefore, continuous surveillance of *P. vivax *and *P. falciparum *molecular markers are needed to monitor the development of resistance to SP in the region.

## Background

*Plasmodium vivax *is responsible for approximately 70-80 million cases of malaria worldwide and causes extensive morbidity in Central and South America and Asia [1]. The extension of geographic distribution of *P. vivax*, the emergence of chloroquine (CQ) resistance [2,3] and also reported fatal cases [4-6] are important issues in developing control strategies. In fact, increasing morbidity and mortality due to emergence of *P. vivax *resistance to CQ [2-6] results in an urgent need to find alternative treatments for *P. vivax *infection, including antifolate drugs.

Molecular studies have been shown that point mutations in the genes that encode dihydrofolate reductase (DHFR) and dihydropteroate synthase (DHPS) enzymes (key enzymes in the biosynthesis and recycling tetrahydrofolate) confer resistance to sulphadoxine-pyrimethamine (SP) in both *Plasmodium falciparum *and *P. vivax *parasites [7-12]. Moreover, the *pvdhfr *and *pvdhps *genotypes might be associated with treatment failure in individual vivax malaria patients [13]. Although data on the genotypes of these two genes are available in Thailand, the Indian subcontinent and the Indonesian archipelago, such data are limited in many regions, most notably Central and South America and the Middle East.

Different investigations showed that mutant alleles of *pvdhfr *gene in areas with a long history of extensive SP use are prevalent; however, wild-type *pvdhfr *has been found more commonly in areas with limited use of SP [8,10,13,14]. So far, over 20 different alleles have been described in *pvdhfr *[15]. Also, different studies of *P. vivax *parasites in various malaria endemic areas, such as Thailand and India showed that mutations at *pvdhfr *codons 57, 58, 61, 117 and 173, [8,16] were found to be involved in clinical antifolate resistance [10,15]. Four mutations have already been identified in *pvdhps *gene at codons 382, 383, 442 and 553 [15,16].

Afghanistan is a country in south-central Asia, where malaria has remained a major public health problem in many of its provinces at altitudes below 2,000 metres with low to high transmission potential. Malaria transmission is seasonal from June to November and the peak for *P. vivax *is around July, but is in October for *P. falciparum*. According to WHO, Afghanistan has the second highest burden of malaria in the Eastern Mediterranean Region (EMR) and the fourth highest rate worldwide, including outside sub-Saharan Africa [17]. National Malaria Control Programmes initiated in 1950s led to a substantial reduction of transmission in Afghanistan, not only by using DDT, but also by increasing the number of diagnosis of malaria patients and treatment. However, the rate of malaria has increased due to war and the lack of the malaria control programme in this country.

After the invasion of the Soviet Union to Afghanistan, the public health system collapsed, health professionals emigrated and poverty increased. Therefore, this resulted in increasing the rates of malaria than any other disease in Afghanistan due to the lack of the malaria control programme. Before the war, in 1970s, the number of recorded cases of malaria per year varied between 40, 000 and 80,000 (an annual incidence of 2.5- 5 per 1000 people). In 2002, the total malaria burden was estimated by the WHO to be 2.2-3 million cases per year. In 2007, 19 cases of malaria per 1,000 population (466,239 total reported cases) were reported; however, the estimated cases by WHO is 1,500,000 [17].

The first detection of *P. falciparum *resistance to chloroquine (CQ) was reported in 1989 and with an increase to 90%, the Ministry of Public Health, National Malaria Control Programme in Afghanistan, decided in 2004 to revise its treatment policy. Therefore, SP-artesunate in combination has been recommended and being used as the first-line anti-malarial treatment, which in fact gives 100% cure rate [17]. CQ plus primaquine remains the first choice drug for treatment of *P. vivax *mono-infections and resistance to either CQ or SP has not been recorded yet [17]. Furthermore, *P. vivax *is sympatric with *P. falciparum *[18] in these areas, but the correct diagnosis of mixed infections based on microscopic examination of blood films is not easy and the clinical symptoms caused by the two species cannot be differentiated. As a result, *P. vivax *populations have often been inadvertently exposed to SP pressure and this may have caused the selection of *P. vivax *SP-resistant isolates.

Molecular markers have been validated as tools for surveillance of resistance; therefore, analysis of *pvdhfr *and *pvdhps *mutations in wild isolates has been considered to be a valuable molecular approach for mapping drug resistance and monitoring malaria control measures. This is the first study to investigate the frequency of SNPs-haplotypes in the *dhfr *and *dhps *genes in *P. vivax *clinical isolates circulating in two malaria endemic areas in Afghanistan. Thus, the out-coming results may be useful for establishing an epidemiological map of drug-resistant vivax malaria, and also updating guidelines for anti-malarial policy in Afghanistan.

## Methods

### Study sites and collection of clinical isolates of *P. vivax*

Blood samples (n = 171) were collected from the patients who were infected with *P. vivax *mono-infection, reported to the health facilities located in Herat and Nangarhar provinces in Afghanistan. Herat, a province in the north-west of Afghanistan on the border between Iran and Turkmenistan, has 15 districts with a population of around 1,182,000. Totally, 233 confirmed cases with *P. falciparum *(n = 3) and *P. vivax *(n = 230) were reported from public health facilities in 2008. Nangarhar is located in the east of Afghanistan on border with Pakistan. The province has 21 districts with a population of around 1,089,000. In 2008, 1352 and 28,823 confirmed cases with *P. falciparum *and *P. vivax *were reported from public health facilities, respectively. The risk of malaria transmission in both areas is moderate to high. After obtaining informed consent from adults or the parents or legal guardians of children, 1 ml of blood was collected from vivax malaria patients on admission, from April to September 2008. All *P. vivax *clinical isolates (Nangarhar = 86 and Herat = 85) were diagnosed by light microscopic examination of Giemsa-stained blood smears. This study was approved by the Ethical Review Committee of Research in Institut Pasteur of Iran.

### Parasite genomic DNA extraction and *pvdhfr/pvdps *genes amplification

Parasite DNA was extracted from 250 μl infected whole blood by phenol/phenol-chloroform extraction and ethanol precipitation as described previously [19]. The DNA was dissolved in 30 μl TE buffer (10 mM Tris-HCL, pH 8.0 and 0.1 mM EDTA). *P. vivax *isolates were genotyped for *dhfr *and *dhps *genes by using previously described PCR-RFLP methods [8,10,20] [Tables 1 and 2].

**Table 1:** Primers and profiles used for amplification of the *pvdhfr *and *pvdhps *genes.

Gene	Nested-PCR (position)	Primer	Sequence			Temperature °C/time (min)				Product size (bp)
					
						A	E	D	C	
*pvdhfr*	Nest-1	VDTOF	ATGGAGGACCTTTCAGATGTATTTGACATT			64 (2')	72 (2')	94 (1')	25	1869
		VDTOR	GGCGGCCATCTCCATGGTTATTTTATCGTG							

	Nest-2 (13, 33, 58, 61)	VDF13NF	GACCTTTCAGATGTATTTGACATTTACGGC			66 (2')	72 (2')	94 (1')	25	232
		VDF13NR	GGTACCTCTCCCTCTTCCACTTTAGCTTCT							

	Nest-2 (57, 117)	VDNF57	CATGGAAATGCAACTCCGTCGATATGATGT			66 (2')	72 (2')	94 (1')	25	472
		VDFNR	TCACACGGGTAGGCGCCGTTGATCCTCGTG							

	Nest-2 (57, 173)	VDTOF	ATGGAGGACCTTTCAGATGTATTTGACATT			66 (2')	72 (2')	94 (1')	25	608
		VDFNR	TCACACGGGTAGGCGCCGTTGATCCTCGTG							

*pvdhps*	Nest-1	VDHPSOF	ATTCCAGAGTATAAGCACAGCACATTTGAG			58 (2')	72 (1')	94 (1')	21	1499
		VDHPSOR	CTAAGGTTGATGTATCCTTGTGAGCACATC							

	Nest-2 (383)	VDHPSNF	AATGGCAAGTGATGGGGCGAGCGTGATTGA			50 (2')	72 (2')	94 (1')	25	703
		VDHPSNR	CAGTCTGCACTCCCCGATGGCCGCGCCACC							

	Nest-2 (553)	VDHPS553OF	TTCTCTTTGATGTCGGCCTGGGGTTGGCCA			68 (1')	72 (1')	94 (1')	30	170
		VDHPSNR	CAGTCTGCACTCCCCGATGGCCGCGCCACC							

**A: **Annealing, **E: **Extension, **D: **Denaturation, **C: **Cycles

**Table 2:** RFLP protocols used for genotyping *pvdhfr *and *pvdhps *genes.

Gene	Primers	RFLP Position	Restriction Enzyme	Company	Uncut Product Size (bp)	Cut Product Size (bp)
*pvdhfr*	VDF13NF/VDF13NR	I13L	*Hae III*	Roche	232	L: 200 + 32
		P33L	*Cfr42I (Sac II)*	Fermentas	232	P: 138 + 94
		S58R	*Alu I*	Fermentas	232	S: 167 + 40 + 25 R: 207 + 25
		T61M	*Tsp45 I*	BioLabs	232	T: 200 + 32
	
	VDTOF/VDFNR	F57I/L	*Xmn I*	Fermentas	608	F: 166 + 442
		I173L	*Eco130 I (Sty I)*	Fermentas	608	L: 438 + 97 + 73 I: 472 + 136
	
	VDNF57/VDFNR	F57I/L	*BsrG I*	BioLabs	472	I: 444 + 28
		S117N/T	*Pvu II*	BioLabs	472	S: 258 + 214
		S117N/T	*Bsr I*	Fermentas	472	N: 219 + 253
		S117N/T	*BstN I*	BioLabs	472	T: 257 + 215

*Pvdhps*	VDHPSNF/VDHPSNR	A383G	*Msp I (Hpa II)*	Fermentas	703	G: 655 + 48
	
	VDHPS553OF/VDHPSNR	A553G	*MscI*	BioLabs	170	A:143 + 27

All amplifications were carried out in a final volume of 25 μl including 1 μl of template from either genomic DNA or the primary reaction. The primers were used at a final concentration of 250 nM and the reaction mixture contained 10 mM Tris-HCL (pH 8.3), 50 mM KCl, 2 mM MgCl_2_, each of the four deoxynucleotide triphosphates at a concentration of 125 μM, and 0.2 U of Taq polymerase (Invitrogen, Carlsbad, CA). The DNA fragments, obtained following PCR amplification or RFLP analysis, were electrophoresed on 2.5% and 3% Metaphor agarose gels (Invitrogen, Carlsbad, CA), respectively.

### Analysis of *pvdhfr *gene at repeat region

The region contains a tandem repeat was amplified using 1 μl of primary reaction with the following primers as described previously [10]:

VDFN2F: CGGTGACGACCTACGTGGATGAGTCAAAGT

VDFN2R: TAGCGTCTTGGAAAGCACGACGTTGATTCT

The cycling conditions for this reaction was 95°C for 5 min, 25 cycles of 66°C for 2 min, 72°C for 2 min, 94°C for 1 min followed by 66°C for 2 min and 72°C for 15 min. The DNA fragments obtained following PCR amplification were analysed following electrophoresis on 3% Metaphor agarose gels. Three size variant types, A (the largest bp), B (the middle bp) and C (the smallest bp), ranging between 230 and 280 bp, were detected in the studied samples.

## Results

### Detection of mutations in the *pvdhfr *and *pvdhps *genes

In this investigation, 171 isolates from Nangarhar and Herat were analysed for SNPs-haplotypes at positions 13, 33, 57, 58, 61, 117 and 173 of the *pvdhfr *and 383 and 553 of *pvdhps *genes and also six *pvdhfr/pvdhps *alleles were identified. In *pvdhfr*, polymorphisms at positions 58R and 117N were found in 2.3% and 13.9% of Nangarhar isolates, respectively. Among Herat isolates, mutations at positions 58R and 117N were found in 2.3% and 3.5% of the studied isolates, respectively (Table 3).

**Table 3:** The frequency distribution of SNPs combinations of *pvdhfr *and *pvdhps *alleles associated with sulphadoxine-pyrimethamine in *P. vivax *isolates from Afghanistan.

*pvdhfr*							*pvdhps*		Nangarhar	Herat
**I13L**	**P33L**	**F57I/L**	**S58R**	**T61M**	**S117N/T**	**I173L**	**A383G**	**A553G**	**n = 86**	**n = 85**

I	P	F	S	T	S	I	A	A	68 (79.1%)	79 (92.9%)
I	P	F	S	T	**N**	I	A	A	10 (11.6%)	1 (1.2%)
I	P	F	S	T	S**N **	I	A	A	3 (3.5%)	3 (3.5%)
I	P	F	**R **	T	**N**	I	A	A	2 (2.3%)	1 (1.2%)
I	P	F	S**R**	T	S	I	A	A	3 (3.5%)	-
I	P	F	**R**	T	**N**	I	**G**	A	-	1 (1.2%)

									**R **= 2.3%	**R **= 2.4%
									S**R **= 3.5%	-
									**N **= 13.9%	**N **= 3.5%
									S**N **= 3.5%	S**N **= 3.5%

In total, all 171 examined isolates were found to carry wild-type amino acids at positions 13, 33, 57, 61 and 173, while 58R and 117N mutations were detected among 4.1% and 12.3% examined samples, respectively (Table 3).

Mixed-genotype infections (58S/R and 117S/N) were both detected in 3.5% (3/86) of Nangarhar isolate but, the mixed-genotype infection (117S/N) was only detected in 3.5% (3/85) of Herat isolates (Table 3).

In the case of *pvdhps *gene, polymorphisms at positions A383G and A553G of *dhps *were investigated and mutation at 383G was only detected in 1.2% (1/85) of Herat samples (Table 3). This mutation was also confirmed by sequencing analysis (accession no. Gu549414).

### Repeat variation in *pvdhfr *gene

**Figure 1:**
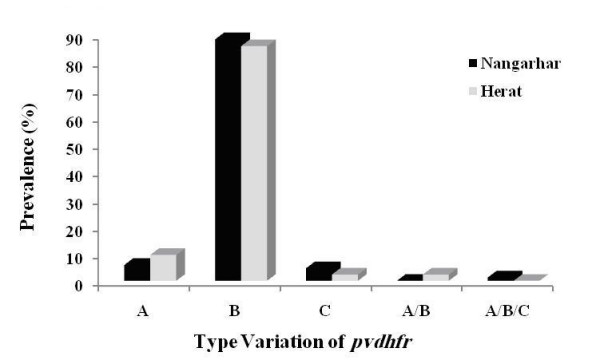
**Frequency distribution of *pvdhfr *repeat region types in wild *P. vivax *isolates from Afghanistan**. **A**: Type A (the largest bp); **B**: Type B (the middle bp); **C**: Type C (the smallest bp); ranging between 230 and 280 bp. **A/B **and **A/B/C**: Mixed genotype infections.

In this investigation, all three types (A, B and C) were found among Afghan isolates. The frequency distribution for type A, B and C were 5.8% (5/86), 88.4% (76/86) and 4.6% (4/86) among Nangarhar isolates, respectively. However, for Herat samples, the prevalence of type A, B and C were 9.4% (8/85), 86% (73/85) and 2.3% (2/85), respectively. Mixed-genotype infections (type A/B) were detected in only 2.3% (2/85) of Herat and mixed types (A/B/C) were detected in 1.2% (1/86) of Nangarhar isolates (Figure 1).

### Comparison of *pvdhfr *and *pvdhps *haplotypes in Afghan isolates with that in other global geographical areas

**Figure 2:**
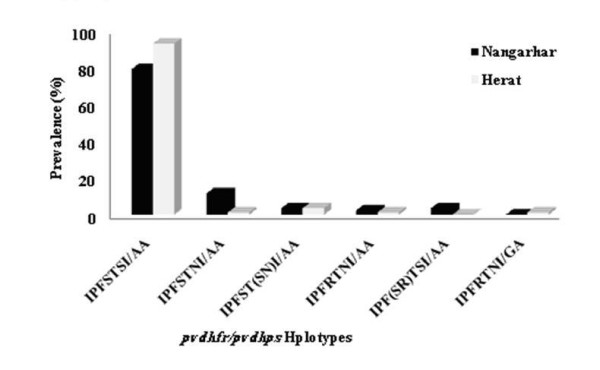
**Frequency distribution of the combination *pvdhfr/pvdhps *haplotypes obtained from 171 isolates collected from Herat province in north-west and Nangarhar province in eastern of Afghanistan**. All six haplotypes are indicated as A to F in the Figure. Mutated amino acids are boldfaced. A) I_13_P_33_F_57_S_58_T_61_S_117_I_173_/A_383_A_553 _B) I_13_P_33_F_57_S_58_T_61_**N**_117_I_173_/A_383_A_553 _C) I_13_P_33_F_57_S_58_T_61_S**N**_117_I_173_/A_383_A_553 _D) I_13_P_33_F_57_**R**_58_T_61_**N**_117_I_173_/A_383_A_553 _E) I_13_P_33_F_57_ S**R**_58_T_61_S_117_I_173_/A_383_A_553 _F) I_13_P_33_F_57_**R**_58_T_61_**N**_117_I_173_/**G**_383_A_553_.

The combination of *pvdhfr *and *pvdhps *haplotypes among all 171 samples in this study demonstrated six distinct haplotypes previously reported from various geographic areas (Figure 2 and Additional file 1). The two most prevalent haplotypes among all examined samples were wild-type (86%) and I_13_P_33_F_57_S_58_T_61_**N**_117_I_173_**/**A_383_A_553 _(6.4%). Alleles with two mutations at position 58R and 117N (I_13_P_33_F_57_**R**_58_T_61_**N**_117_I_173_/A_383_A_553_) accounted for 1.7% of the total isolates and I_13_P_33_S_57_**R**_58_T_61_**N**_117_I_173_/**G**_383_A_553 _(1.2%) mutant haplotype was only detected among Herat but in none of the Nangarhar isolates (Figure 2 and Table 3). In addition, based on size polymorphism of *pvdhfr *genes at repeat region, type B was identified at high proportion in Afghan *P. vivax *isolates and I_13_P_33_F_57_**R**_58_T_61_**N**_117_I_173_/A_383_A_553 _and I_13_P_33_S_57_**R**_58_T_61_**N**_117_I_173_/**G**_383_A_553 _mutant haplotypes were B type (Table 4).

**Table 4:** Association between mutations and tandem repeat region type of *pvdhfr *gene in *P. vivax *isolates from Afghanistan.

Repeat region type	*pvdhfr*							*pvdhps*		Nangarhar	Herat
			
	I13L	P33L	F57I/L	S58R	T61M	S117N/T	I173L	A383G	A553G	n = 86	n = 85
**A**	I	P	F	S	T	**N**	I	A	A	2 (2.3%)	-
	I	P	F	S	T	S	I	A	A	3 (3.5%)	8 (9.4%)

**B**	I	P	F	**R**	T	**N**	I	A	A	2 (2.3%)	1 (1.2%)
	I	P	F	S	T	**N**	I	A	A	8 (9.3%)	1(1.2%)
	I	P	F	S	T	S	I	A	A	61 (71%)	67 (78.8%)
	I	P	F	S**R**	T	S	I	A	A	3 (3.5%)	-
	I	P	F	S	T	S**N**	I	A	A	2 (2.3%)	3 (3.6%)
	I	P	F	S	T	**N**	I	**G**	A	-	1(1.2%)

**C**	I	P	F	S	T	S	I	A	A	4 (4.6%)	2 (2.3%)

**A/B**	I	P	F	S	T	S	I	A	A	-	2 (2.3%)

**A/B/C**	I	P	F	S	T	S**N**	I	A	A	1 (1.2%)	-

The most common mutant haplotype is 58R/117N of *pvdhfr *reported from other areas including Iran [21], Pakistan [22], Thailand [23], India [24], Philippines, Vietnam, East Timor, PNG, Vanuatu and Vietnam [25], Colombia [15], Myanmar [26] and Madagascar [16], but not from Azerbaijan and Turkey [27].

So far, quadruple mutant alleles of *pvdhfr *that are associated with SP treatment failure were reported from Myanmar [26], India [24], PNG, Vanuatu [25], and Thailand [23] but not from other geographic areas (Additional file 1). Double (383G/553G) and triple (382A/383G/553G) mutant alleles of *pvdhps *were only reported from Thailand [23] (Additional file 1).

## Discussion

Efforts toward controlling malaria are greatly challenged by the increasing spread of anti-malarial drug resistance and also the use of ineffective anti-malarial drugs. Therefore, there is a need for monitoring anti-malarial drug efficacy and drug resistance in global malaria endemic regions. In the present study, for the first time, the prevalence of mutations in the SP resistance-associated genes, *dhfr *and *dhps *was determined in 171 blood samples infected with *P. vivax *collected from two malaria endemic areas of Afghanistan where both CQ and SP were used for treatment. Vivax infections are not often treated with SP; therefore, *P. vivax *isolates are exposed to SP because mixed infections are present in these regions and are often mis-diagnosed [18].

Afghanistan is a country in south-central Asia that bordered to the west with Iran and to the east and south with Pakistan. Due to war, population displacements and movements across the borders have also occurred and this might contribute to the spread of disease and also parasite resistance to anti-malarial drugs to neighbouring countries. Although there is no *in vivo *evidence of *P. vivax *resistance to CQ in Afghanistan, an *in vivo *work in Iran in 2005 [28] showed that parasite clearance time increased when compared to 2001 in Sistan and Baluchistan province. This indicates that it could be an early sign of reduced susceptibility of the *P. vivax *populations to CQ in these regions. Therefore, an effective alternative drug against *P. vivax *resistance to CQ might be needed in near future.

In the present investigation, a limited polymorphism in *pvdhfr *and *pvdhps *genes has been detected that was in contrast with earlier studies in Myanmar [26], PNG, Vanuatu [25], and Thailand [23]. In total, six distinct haplotypes of *pvdhfr *were detected among Afghan isolates. The wild-type *dhfr/dhps *haplotype is present at high proportion in *P. vivax *parasite populations from both study areas in Afghanistan, which was similar to that of obtained from malaria endemic regions in Iran [21] and Pakistan [22]. The single mutant I_13_P_33_F_57_S_58_T_61_**N**_117_I_173/_A_383_A_553 _(6.4%) was the second frequent haplotype in Afghan *P. vivax *isolates; however, double mutant I_13_P_33_F_57_**R**_58_T_61_**N**_117_I_173/_A_383_A_553 _was the second frequent haplotype in Iranian (9.5%) and Pakistani (16.1%) isolates [21,22]. The explanation for low prevalence of I_13_P_33_F_57_**R**_58_T_61_**N**_117_I_173_/A_383_A_553 _haplotype (1.7%) among Afghan isolates may be due to the recent usage of SP as the first-line anti-malarial treatment in these areas, as different studies revealed that wild-type *pvdhfr *has been found more commonly in areas with limited use of SP [8,10,13,14]. Moreover, the frequency distribution of *pvdhfr *mutant haplotypes was significantly higher in the Nangarhar (20.9%) than Herat province (7%). This might be also due to gene flow of SP resistance in *P. vivax *populations in a consequence of human migration across border between Pakistan and Afghanistan in Nangarhar province.

Based on size polymorphism of *pvdhfr *gene at repeat region, among Afghan isolates, type B was identified at high proportion in both study areas similar to the findings from its neighbouring country, Iran [21]. The present investigation also showed the association between mutant haplotypes and type B in both study areas; however final conclusion for such association needs further study in global vivax malaria endemic region. In addition, mixed genotype infections, types A/B and A/B/C were detected in Herat and Nangarhar isolates, respectively; however, the only mixed type detected in Iranian malaria settings was B/C genotype [21].

The 58R and 117N were found in 4.1% and 12.3% of all examined isolates, respectively and in combination with each other (58R/117N) in 2.3%. Surprisingly, 117N was detected at high frequency among isolates collected from Turkey (36%), Azerbaijan (71%) and Pakistan (93.5%) [22,27].

Mutations at codons 58 and 117 in *pvdhfr *gene are also considered to be equivalent to mutations at residues 59 and 108 in *pfdhfr*, respectively that are known to be associated with pyrimethamine resistance. In fact, double mutations at codons 58R and 117N in *pvdhfr *may arise first under drug pressure and move toward the development of resistance to SP [8]. As a result, these two mutations were detected in *P. vivax *populations in Iran [21], Pakistan [22] and Afghanistan [present study] three years after using SP-artesunate as first-line treatment of uncomplicated *P. falciparum *in these regions. In the present study, quadruple mutants were not detected among examined isolates; however, quadruple mutant alleles of *pvdhfr *at codons 57, 58, 61 and 117 predominated in clinical isolates in Thailand, where *P. falciparum *showed multi-drug resistance [23], Myanmar, Indonesia and India [8,10,26,29,30]. The difference in the prevalence of mutant *pvdhfr *alleles reflects the selection pressure exerted by usage of the antifolate drug in these countries.

The work carried out by Tahar and colleagues [31] showed that the 58R/117N mutant had a lower affinity for pyrimethamine and cycloguanil than did the wild-type enzyme. Different studies also showed that in areas where antifolate has been intensively used, such as Thailand and Indonesian Papua, haplotypes that carry more than two mutations of *dhfr *are more prevalent and surely are resistance to pyrimethamine [8,10,14,27,32]. Patients whose parasites carried the 57L/61M/117T/173F allele were more likely to fail SP treatment [14,29]. In addition, treatment failure was more frequently associated with multiple mutations in *pvdhfr *and *pvdhps *[20] and also when the parasite carries mutant alleles of both genes, clinical effectiveness is compromised [33-35]. In the present study, the most common haplotypes of *pvdhfr *were wild-type and double mutant (58R and 117N), quadruple mutant were not detected among examined isolates. This suggests that a DHFR inhibitor could be effective in treatment against the erythrocytic stages of vivax malaria. In contrast, molecular analysis of *pvdhfr *among Indian field isolates showed haplotypes from wild-type to quadruple mutant genotype. These haplotypes may be come from Indian subcontinent to this area as gene flow of anti-malarial drug resistance in malaria parasites might be often a consequence of human migration rather than the emergence of new mutations. Moreover, the results support the concept of east to west reduction in SP pressure and this might be reflected in the presence of different mutations in the *pvdhfr *gene.

## Conclusions

The present molecular data shows a limited polymorphism in *pvdhfr *from Afghan isolates and it provides important basic information for monitoring SP resistance in Afghanistan. The present study shows high prevalence of wild-type and low frequency of single mutated *pvdhfr *alleles among Afghan isolates. However, the continuous SP pressure in Afghanistan might progress mutations in the *dhfr *gene in both *P. vivax *and *P. falciparum *species, which may finally lead to a complete SP resistance in this region. Therefore, continuous surveillance of *P. vivax *and *P. falciparum *molecular markers are needed to monitor the development of resistance to SP. Moreover, such information is necessary for guiding malaria control measures in the frame of Roll Back Malaria strategies for eliminating of malaria in this region.

## Competing interests

The authors declare that they have no competing interests.

## Authors' contributions

SZ designed and supervised the study, analysed the data and wrote the manuscript. MA and FGH contributed in the laboratory work and helped with analysis of the data. AR, NS, WB, and HA participated in field work, study coordination and preliminary analysis. NDD helped with analysis of the data and also helped with the writing of the manuscript. All authors read and approved the final manuscript.

## Supplementary Material

Additional file 1 Frequency distribution of different haplotypes of *pvdhfr and pvdhps *in different geographic areas. Click here for file
