# Supplementary material for: Molecular surveillance of Plasmodium vivax dhfr and dhps mutations in isolates from Afghanistan
Source: Malar J. 2010 Mar 14;9:75. doi: 10.1186/1475-2875-9-75 (PMC2848684; doi:10.1186/1475-2875-9-75)
Supplement: Additional file 1 — Frequency distribution of different haplotypes of pvdhfr and pvdhps in different geographic areas. [file 1475-2875-9-75-S1.DOC]

**Additional file 1**: Frequency distribution of different haplotypes of *pvdhfr and pvdhps* in different geographic areas.

| **Study Site** | ***pvdhfr* alleles** | ***pvdhps* allele** | **No (%)** | **References** |
| --- | --- | --- | --- | --- |
| **Afghanistan (n = 171)** | Wild-type | Wild -type | 147 (86) | Present Study |
|  | 117N | Wild -type | 11 (6.4) |  |
|  | 117S/N | Wild -type | 6 (3.6) |  |
|  | 58R+117N | Wild -type | 3 (1.7) |  |
|  | 58S/R | Wild -type | 3 (1.7) |  |
|  | 58R+117N | Wild -type | 1 (0.6) |  |
|  |  |  |  |  |
| **Iran**  **(n = 189)** | Wild Type | Wild -type | 124 (65.6) | [21] |
|  | 117N | Wild -type | 31 (16.4) |  |
|  | 58R | Wild -type | 14 (7.4) |  |
|  | 58R+117N | Wild -type | 18 (9.6) |  |
|  | 57L+58R | Wild -type | 1 (0.5) |  |
|  | 57L | Wild -type | 1 (0.5) |  |
|  |  |  |  |  |
| **Pakistan**  **( n = 95)** | 57L+58R | Wild -type | (1.7) | [22] |
|  | 58R+117N | Wild -type | (16.1) |  |
|  |  |  |  |  |
| **Azerbaijan**  **( n = 39)** | Wild Type | - | 25 (64) | [27] |
|  | 117N | - | 14 (35.8) |  |
|  |  |  |  |  |
| **Turkey**  **(n = 24)** | Wild Type | - | 7 (29) | [27] |
|  | 117N | - | 17 (70.8) |  |
|  |  |  |  |  |
| **India**  **( n = 121)** | Wild Type | - | 85 (70) | [24] |
|  | 109H | - | 3 (2.5) |  |
|  | 58R+117N | - | 16 (13) |  |
|  | 57L+58R+61M+117T | - | 2 (1.65) |  |
|  | 58R | - | 2 (1.65) |  |
|  | 57L+58R | - | 2 (1.65) |  |
|  | 57L+58R+109C | - | 5 (4) |  |
|  | 58R+117N+188V | - | 1 (0.8) |  |
|  | 58R+117N+159A | - | 1 (0.8) |  |
|  | 58R+117N+171L | - | 1 (0.8) |  |
|  | 38G+58R+109H+159A | - | 1 (0.8) |  |
|  | 117N | - | 1 (0.8) |  |
|  | 131G | - | 1 (0.8) |  |
|  |  |  |  |  |
| **Thailand**  **(n = 32)** | No Wild Type | No Wild -type |  | [23] |
|  | 58R+117N | 383G+553G | 1 (3.1) |  |
|  |  | 383G | 1 (3.1) |  |
|  | 49C+58R+117N | 383G | 1 (3.1) |  |
|  | 58R+61M+117T | 383G+553G | 3 (9.4') |  |
|  | 57L+58R+61M+117+T | 383G+553G | 8 (25) |  |
|  |  | 382A+383G+553G | 1 (3.1) |  |
|  | 57I+58R+61M+117T | 383G+553G | 12 (37.5) |  |
|  |  | 382A+383G+553G | 3 (9.4) |  |
|  |  | 382C+383G+553G | 2 (6.3) |  |
|  |  |  |  |  |
| **China**  **( n = 7)** | Wild Type | Wild -type | 5 (71.4) | [25] |
|  | 117N | Wild -type | 1 (14.3) |  |
|  | 58R+117N | Wild -type | 1 (14.3) |  |
| **Philippines**  **( n = 15)** | Wild Type | Wild -type | 5 (33.3) | [25] |
|  | 58R+117N | 383A/G | 10 (66.7) |  |
|  |  |  |  |  |
| **Vietnam**  **( n = 7)** | Wild Type | Wild -type | 2 (28.6) | [25] |
|  | 58R | Wild -type | 1 (14.3) |  |
|  | 58R+117N | 383G | 4 (57.1) |  |
|  |  |  |  |  |
| **Myanmar**  **( n = 18)** | Wild Type | - | 1 (5.5) | [26] |
|  | 58R+117N | - | 9 (50) |  |
|  | 58R+117T | - | 3 (16.6) |  |
|  | 57L+58R+61M+117N | - | 3 (16.6) |  |
|  | 57L+58R+61M+117T | - | 2 (11.1) |  |
|  | 57I+58R+61M+117T | - | 3 (16.6) |  |
|  |  |  |  |  |
| **East Timor ( n = 18)** | Wild Type | Wild -type | 4 (22.2) | [25] |
|  | 58R | Wild -type | 1 (5.6) |  |
|  | 117N | Wild -type | 3 (16.7) |  |
|  | 58R+117N | Wild -type | 10 (55.6) |  |
|  |  |  |  |  |
| **PNG**  **( n = 5)** | Wild Type | Wild -type | 1 (20) | [25] |
|  | 57L+58R | Wild -type | 1 (20) |  |
|  | 57L+117T | Wild -type | 1 (20) |  |
|  | 58R+117N | Wild -type | 1 (20) |  |
|  | 57L+58R+61M+117T | 553A/G | 1 (20) |  |
|  |  |  |  |  |
| **Vanuatu**  **( n = 18)** | Wild Type | Wild -type | 1 (5.6) | [25] |
|  | 58R+117N | Wild -type | 4 (8.3) |  |
|  | 58R+117T | Wild -type | 1 (5.6) |  |
|  | 58R+61M+117T | Wild -type | 10 (55.6) |  |
|  | 57L+117T+173F | Wild -type | 1 (5.6) |  |
|  | 57F/L+58R+61M+117T+173F | Wild -type | 1 (5.6) |  |
|  |  |  |  |  |
| **Madagascar**  **( n = 159)** | Wild Type | Wild -type | 44 (27.7) | [16] |
|  |  | 383G | 2 (1.3) |  |
|  | 21S | Wild -type | 2 (1.3) |  |
|  | 33L | Wild -type | 8 (5) |  |
|  | 117N | Wild -type | 7 (4.4) |  |
|  | 130K | Wild -type | 1 (0.6) |  |
|  | 33L+130k | Wild -type | 1 (0.6) |  |
|  | 117N+130K | Wild -type | 2 (1.3) |  |
|  | 58R+117N | Wild -type | 25 (15.7) |  |
|  |  | 383G | 16 (10.1) |  |
|  | 49R+58R+117N | Wild -type | 44 (27.6) |  |
|  |  | 383G | 4 (2.5) |  |
|  | 58R+117N+130K | 422R | 1 (0.6) |  |
|  |  | 383G | 2 (1.3) |  |
|  |  |  |  |  |
| **Colombia**  **(n = 8)** | 58R+117N | - | 8 | [15] |

- indicate that *dhps* gene was not analysed.
